# Supplementary material for: Wolbachia incompatible insect technique program optimization over large spatial scales using a process-based model of mosquito metapopulation dynamics
Source: BMC Biol. 2024 Nov 21;22:269. doi: 10.1186/s12915-024-02070-1 (PMC11580355; doi:10.1186/s12915-024-02070-1)
Supplement: Supplementary file 1 — Additional file 1: Figure S1, Tables S1 and S2. Figure S1 – Equations for the process-based model. Table S1 – Process-based model parameters. Table S2 – Process-based model functions. [file 12915_2024_2070_MOESM1_ESM.docx]

#### **Additional File 1**


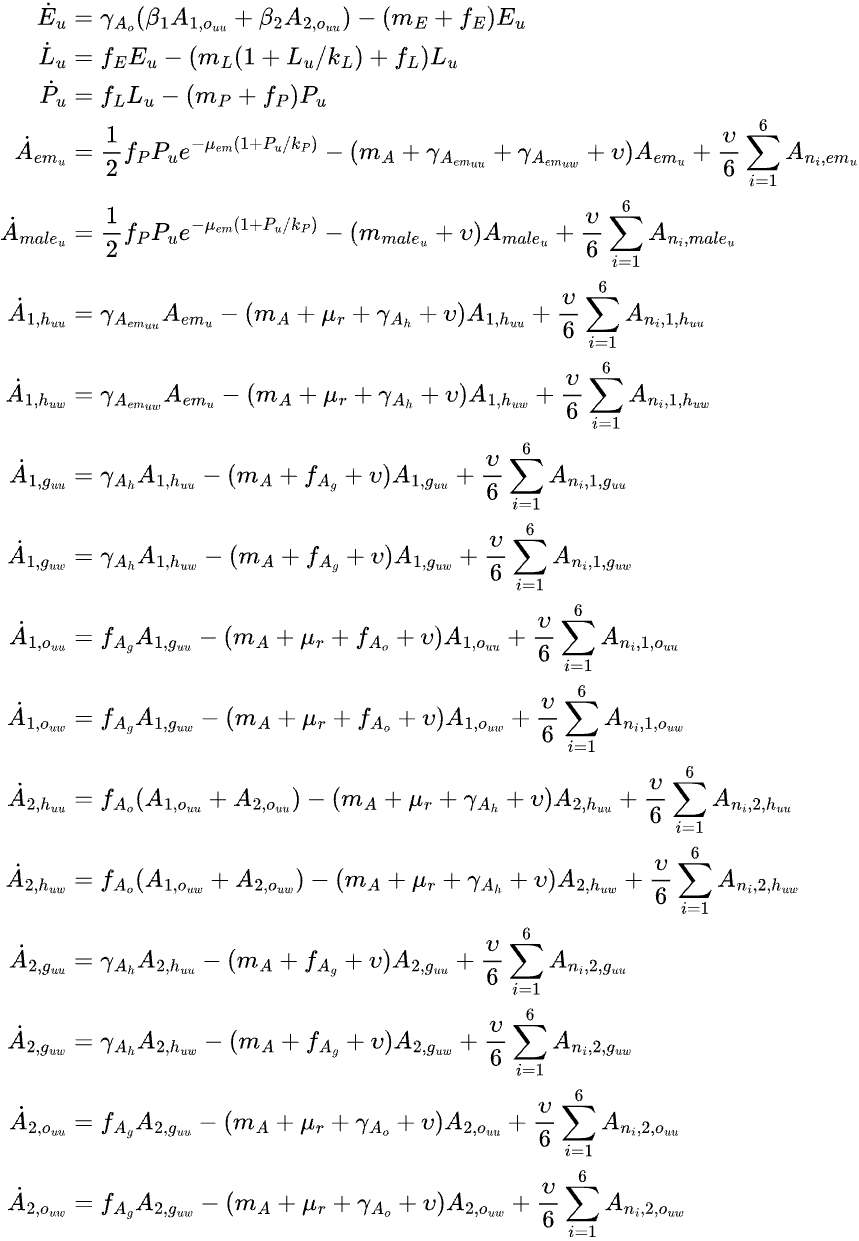


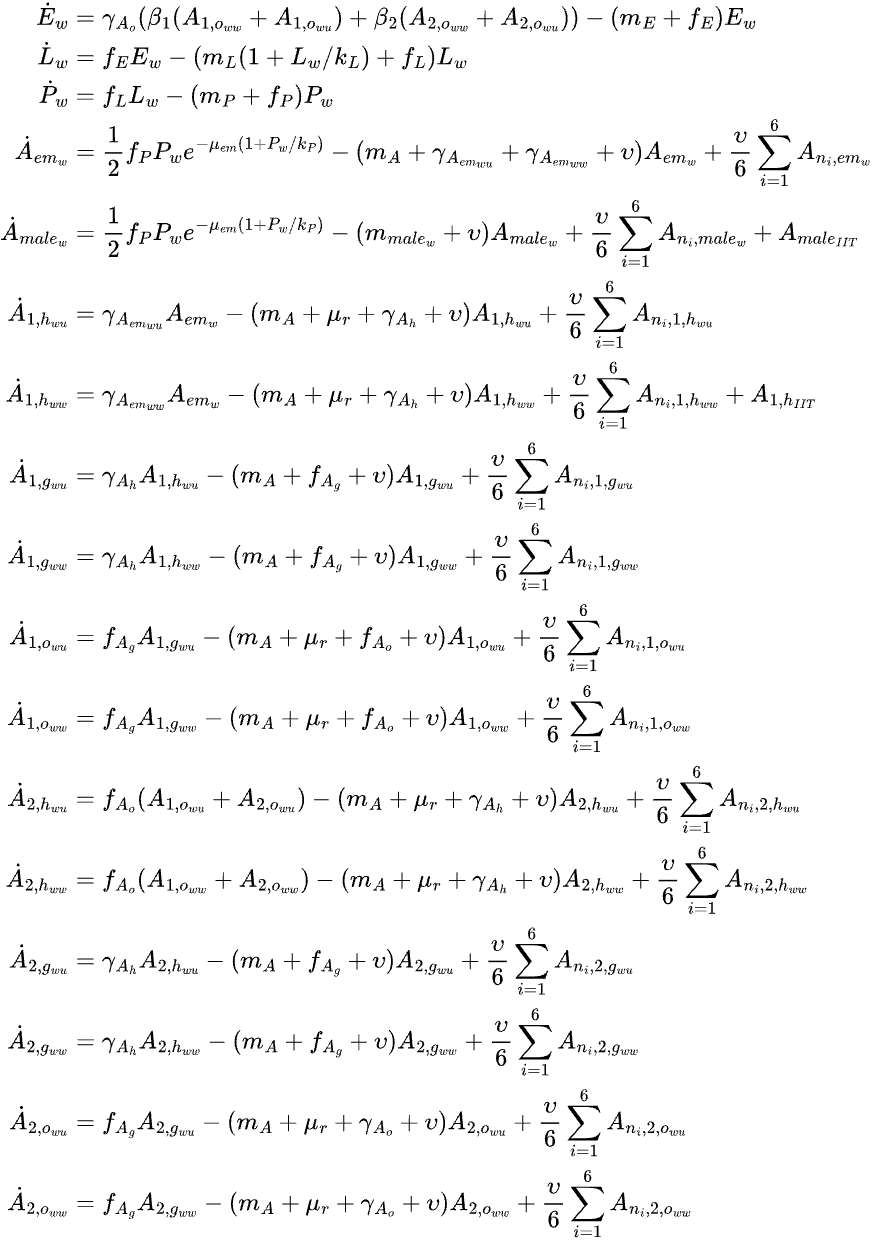


where


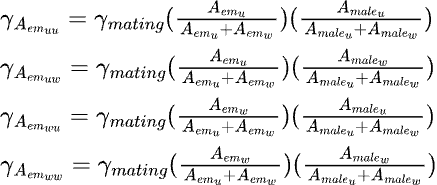


**Figure S1.** Equations for the process-based model.

**Table S1.** Process-based model parameters.

| **Notation** | **Definition** | **Value** | **Reference** |
| --- | --- | --- | --- |
| *β_1_* | Number of eggs laid per ovipositing nulliparous female | 60.0 | (42) |
| *β_2_* | Number of eggs laid per ovipositing parous female | 80.0 | (42) |
| 𝛾*_mating_* | Transition rate from emergent to host-seeking adults per day | 0.4 | (42) |
| 𝛾*_Ah_* | Transition rate from host-seeking to engorged adults per day | 0.2 | (42) |
| 𝛾*_Ao_* | Minimum transition rate from ovipositing to host-seeking adults per day | 0.2 | (42) |
| *µ*_E_ | Minimum egg mortality rate per day | 0.01 | (42) |
| *µ*_em_ | Mortality rate during emergence per day | 0.1 | (42) |
| *µ*_r_ | Mortality rate related to seeking behavior per day | 0.08 | (42) |
| *k_Lfix_* | Maximum human-dependent larval carrying capacity index in a specific hexagon | 100 | Reference index |
| *k_Pfix_* | Maximum human-dependent larval carrying capacity index in a specific hexagon | 100 | Reference index |
| *k_Lvar_* | Maximum rainfall-dependent larval carrying capacity index in a specific hexagon | 50 | Derived^1^ |
| *k_Pvar_* | Maximum rainfall-dependent pupal carrying capacity index in a specific hexagon | 50 | Derived^1^ |
| 𝜐 | Migration rate of adult mosquitoes out of a hexagon per day | 0.0663 | Derived^2^ |
| 𝜂_maleu_ | Adult male mortality factor (relative to adult female mosquitoes) | 2 | Derived^3^ |
| 𝜂_malew_ | Additional male *Wolbachia*-infected mortality factor (relative to adult male mosquitoes) | 0.3 | Derived^4^ |

Details can be found in the following sub-sections of Additional File 2:

^1^*Derivation of the relative ratio between fixed and variable carrying capacities*

^2^*Derivation of the migration rate*

^3^*Derivation of the adult male mortality factor*

^4^*Derivation of the additional adult Wolbachia-infected male mortality factor*

**Table S2.** Process-based model functions.

| **Notation** | **Definition** | **Expression** | **Reference** |
| --- | --- | --- | --- |
| *f_E_* | Transition function from egg to larva | _A_R(1298-1TK)1 + e_HH_R(1T1/2H-1TK)  $\frac{24\rho\frac{T^{K}}{298}e^{\frac{\Delta H_{A}}{R}\left( \frac{1}{298}-\frac{1}{T^{K}} \right)}}{1+e^{\frac{\Delta H_{H}}{R}\left( \frac{1}{T_{1/2H}}-\frac{1}{T^{K}} \right)}}$  Where $\rho$= 0.01066; ΔH_A_ = 10,798.18; ΔH_H_ = 100,000; T_1/2H_ = 14,184.5 | (42) |
| *f_L_* | Transition function from larva to pupa | $\frac{24\rho\frac{T^{K}}{298}e^{\frac{\Delta H_{A}}{R}\left( \frac{1}{298}-\frac{1}{T^{K}} \right)}}{1+e^{\frac{\Delta H_{H}}{R}\left( \frac{1}{T_{1/2H}}-\frac{1}{T^{K}} \right)}}$  Where $\rho$= 0.00873; ΔH_A_ = 26,018.51; ΔH_H_ = 55,990; T_1/2H_ = 304.58 | (42) |
| *f_P_* | Transition function from pupa to emergent adult | $\frac{24\rho\frac{T^{K}}{298}e^{\frac{\Delta H_{A}}{R}\left( \frac{1}{298}-\frac{1}{T^{K}} \right)}}{1+e^{\frac{\Delta H_{H}}{R}\left( \frac{1}{T_{1/2H}}-\frac{1}{T^{K}} \right)}}$  Where $\rho$= 0.0161; ΔH_A_ = 14,931.94; ΔH_H_ = -472,379; T_1/2H_ = 148.45 | (42) |
| *f_Ag_* | Transition function from engorged adult to ovipositing site-seeking female adult | $\frac{24\rho\frac{T^{K}}{298}e^{\frac{\Delta H_{A}}{R}\left( \frac{1}{298}-\frac{1}{T^{K}} \right)}}{1+e^{\frac{\Delta H_{H}}{R}\left( \frac{1}{T_{1/2H}}-\frac{1}{T^{K}} \right)}}$  Where $\rho$= 0.00898; ΔH_A_ = 15,725.23; ΔH_H_ =1,756,481.07; T_1/2H_ = 447.17 | (42) |
| *f_Ao_* | Transition function from ovipositing adult to host-seeking female adult | 𝛾_Ao_ × (1+*P*_norm_) | (42) |
| *m_E_* | Egg mortality rate | *µ_E_* +0.1 if *P* > 80,  *µ_E_* otherwise | (42) |
| *m_L_* | Larval mortality rate | 0.52 + 0.0007e^0.1838(T-10)^ if *P* > 80,  0.02 + 0.0007e^0.1838(T-10)^ otherwise | (42) |
| *m_P_* | Pupal mortality rate | 0.52 + 0.0003e^0.2228(T-10)^ if *P* > 80,  0.02 + 0.0003e^0.2228(T-10)^ otherwise | (42) |
| *m_A_* | Adult female mortality rate | 0.025 + 0.0003e^0.1745(T-10)^ | (42) |
| *m_maleu_* | Adult uninfected male mortality rate | 𝜂*_maleu_* × *m_A_* | Derived^1^ |
| *m_malew_* | Adult *Wolbachia*-infected male mortality rate | (1+ 𝜂*_malew_*) × *m_maleu_* | Derived^2^ |
| *k_L_* | Total larval carrying capacity index per hexagon | *k_L_* = *H_norm_ k_Lfix_ + P_norm_ k_Lvar_* | Derived^3^ |
| *k_P_* | Total pupal carrying capacity index per hexagon | *k_P_ = H_norm_ k_Pfix_ + P_norm_ k_Pvar_* | Derived^3^ |

Details can be found in the following sub-sections of Additional File 2:

^1^*Derivation of the adult male mortality factor*

^2^*Derivation of the additional adult Wolbachia-infected male mortality factor*

^3^*Derivation of the carrying capacity index functions*
