# Supplementary material for: Wolbachia incompatible insect technique program optimization over large spatial scales using a process-based model of mosquito metapopulation dynamics
Source: BMC Biol. 2024 Nov 21;22:269. doi: 10.1186/s12915-024-02070-1 (PMC11580355; doi:10.1186/s12915-024-02070-1)
Supplement: Supplementary file 2 — Additional file 2: Parameter derivations and Figures S2 and S3. Figure S2 – Derived probability distribution of mosquitoes leaving a hexagon. Figure S3 – Carrying capacities for hexagons. [file 12915_2024_2070_MOESM2_ESM.docx]

#### **Additional File 2**

**Parameter derivation**

**Derivation of the relative ratio between fixed and variable carrying capacities** We assumed the relative ratio between human- to precipitation- dependent carrying capacities to be 2:1 based on a Singapore National Environmental Agency report of the number of mosquito breeding sites in residential and non-residential areas (44). Specifically, we assumed that breeding sites found in homes were human-dependent and those not in homes were precipitation-dependent.

**Derivation of migration rate**

The Singapore National Environmental Agency reported that almost all male IIT mosquitoes were caught within 40 meters from where they were released, and that the mosquitoes had a median post-release lifespan of 4 days (45). From this, we assumed that the mean horizontal travel distance for all IIT mosquitoes was 40 meters, and determined that the mean lifespan was 5.77 days by using the following formula, assuming that mosquito longevity followed an exponential decay:

Mean lifespan = half life / log (2)

We then derived the mean daily horizontal travel distance by assuming that mosquito dispersal followed a 2D Gaussian distribution, where the average distance from the mean over 5.77 days was 40 meters. The single-day horizontal travel distance of 16.65 meters was derived as such:

40 meters = $\sqrt{5.77\left( \sigma_{x}^{2}+\sigma_{y}^{2} \right)}$Daily average distance = $\sqrt{\sigma_{x}^{2}+\sigma_{y}^{2}}$

= 16.65 meters

The probability of a mosquito leaving the hexagon was determined by running a simulation with 1000 iterations, where in each iteration, a random point within the hexagon corresponding to the initial mosquito position and a random direction representing the single-day direction of travel was selected. The single-day probability that a mosquito would leave a hexagon was the proportion of iterations where the final mosquito position was outside of the bounds of the hexagon. This simulation was repeated 1000 times, and a mean value of 6.63% was obtained.

We assumed this migration rate applied to all adult mosquitoes in this study.


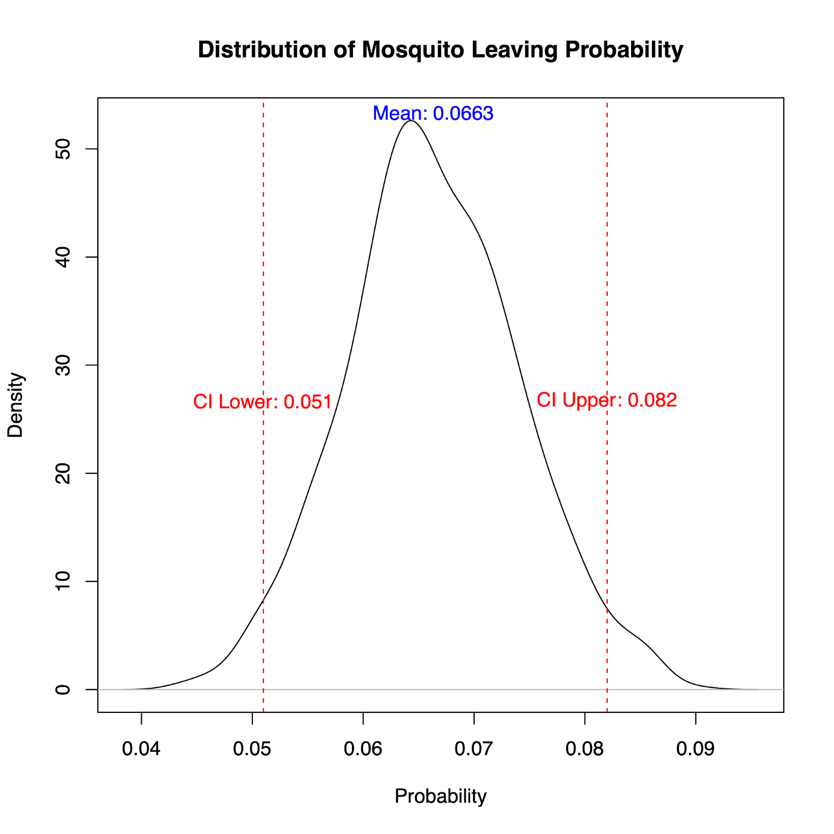


**Figure S2. Derived probability distribution of mosquitoes leaving a hexagon.**

**Derivation of the adult male mortality factor** Magori et al. (26) determined that the adult male mortality probability was about twice that of adult females based on field estimates. Therefore, we define the relative adult male mortality factor, 𝜂_maleu_, to be 2. This factor is applied to the adult female mortality rate function to obtain the adult uninfected male mortality rate function.

Given that the adult female mortality rate function as described by Bonnin et al. (42) is:

*m_A_* =0.025 + 0.0003e^0.1745(T-10)^

The adult uninfected male mortality rate function, *m_maleu_*, is then:

*m_maleu_* = 𝜂*_maleu_* × *m_A_*

**Derivation of the additional adult *Wolbachia*-infected male mortality factor** Ant et al. (46) conducted fitness assessments of *wAlbB Wolbachia*-infected and uninfected male *Aedes aegypti* mosquitoes and found that the median survival times were approximately 30 and 39 days respectively. Therefore, we estimate the average daily mortality rate to be:

Daily mortality rate = 1 / median survival

We define the additional male *Wolbachia*-infected mortality factor, 𝜂*_malew_*, to be:

𝜂*_malew_* = (Daily mortality rate*_wAlbB_* - Daily mortality rate*_u_*) / Daily mortality rate*_u_*

= (1/30 - 1/39) / (1/39)

= 0.3

This additional male *Wolbachia*-infected mortality factor, 𝜂*_malew_*, of 0.3 is then applied to the adult uninfected male mortality rate function to obtain the adult *Wolbachia*-infected male mortality rate.

Given that the adult uninfected male mortality rate function, *m_maleu_*, is:

*m_maleu_* = 𝜂*_maleu_* × *m_A_*

The adult *Wolbachia*-infected male mortality rate function, *m_malew_*, is then:

*m_malew_* = (1+𝜂*_malew_*) × *m_maleu_*

**Derivation of the carrying capacity index functions**

Tran et al. (43) defined the carrying capacity for larvae and pupae respectively over time with the following equations:

*k_L_ = k_Lfix_ + P_norm_ k_Lvar_*

*k_P_ = k_Pfix_ + P_norm_ k_Pvar_*

*P_norm_* is the amount of cumulative rainfall over one week and normalized to vary from 0 to 1. The equations indicate that the carrying capacity for each stage is driven by a fixed human-dependent component (*k_Xfix_*) as well as a precipitation-dependent one (*k_Xvar_*).

In order to account for the varying population densities across different residential areas in Singapore, we modified the equations to incorporate a normalized human population term, *H_norm_*. *H_norm_* is defined as the number of people living in the subzone in which the center of the hexagon falls, normalized against the most populated subzone to vary from 0 to 1. The modified equations are as follows:

*k_L_* = *H_norm_ k_Lfix_ + P_norm_ k_Lvar_*

*k_P_ = H_norm_ k_Pfix_ + P_norm_ k_Pvar_*

Figure S3 shows the carrying capacities of the hexagons across the various simulations. Note that the larval carrying capacity is the same as the pupal carrying capacity in this study.


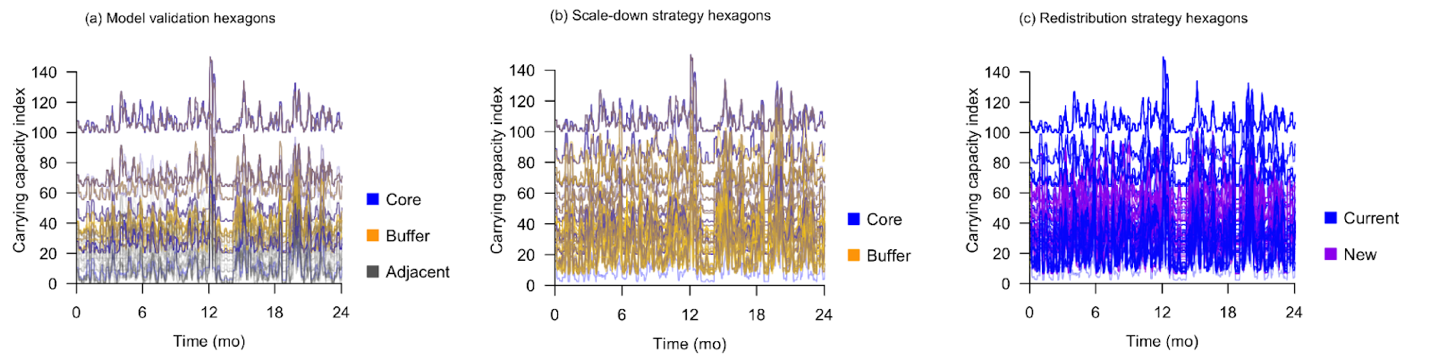


**Figure S3.** **Carrying capacities for hexagons in the (a) model validation, (b) scale-down strategy, and (c) redistribution strategy simulations respectively.**
