# Supplementary material for: Wolbachia incompatible insect technique program optimization over large spatial scales using a process-based model of mosquito metapopulation dynamics
Source: BMC Biol. 2024 Nov 21;22:269. doi: 10.1186/s12915-024-02070-1 (PMC11580355; doi:10.1186/s12915-024-02070-1)
Supplement: Supplementary file 3 — Additional file 3: Weather station list. [file 12915_2024_2070_MOESM3_ESM.docx]

#### **Additional File 3**

**Weather station list**

List of weather stations used for daily precipitation data: S06, S07, S08, S23, S24, S25, S33, S35, S40, S43, S44, S50, S60, S61, S64, S66, S69, S71, S77, S78, S79, S80, S81, S82, S84, S88, S89, S90, S94, S104, S106, S107, S108, S109, S111, S112, S113, S114, S115, S116, S117, S118, S119, S120, S123.

List of weather stations used for daily mean temperature data: S06, S24, S43, S44, S50, S60, S104, S106, S107, S108, S109, S111, S115, S116, S117.
