# Supplementary material for: Wolbachia incompatible insect technique program optimization over large spatial scales using a process-based model of mosquito metapopulation dynamics
Source: BMC Biol. 2024 Nov 21;22:269. doi: 10.1186/s12915-024-02070-1 (PMC11580355; doi:10.1186/s12915-024-02070-1)
Supplement: Supplementary file 4 — Additional file 4: Tables S3-5. Table S3 – List of subzones in the model validation study. Table S4 – List of subzones in the scale-down strategies study. Table S5 – List of new subzones in the redistribution strategies study. [file 12915_2024_2070_MOESM4_ESM.docx]

#### **Additional File 4**

**Table S3. List of subzones in the model validation study.**

Model validation subzones have a total population of 566,990.

| **Zone** | **Subzone** | **Population size** |
| --- | --- | --- |
| Tampines | Tampines North | 25680 |
| Tampines | Tampines East | 126460 |
| Tampines | Tampines West | 81370 |
| Yishun | Yishun West | 52160 |
| Yishun | Yishun Central | 2960 |
| Yishun | Yishun South | 41920 |
| Yishun | Yishun East | 70190 |
| Yishun | Northland | 27280 |
| Choa Chu Kang | Yew Tee | 40070 |
| Choa Chu Kang | Peng Siang | 34250 |
| Choa Chu Kang | Keat Hong | 38990 |
| Bukit Batok | Hong Kah North | 25660 |

**Table S4. List of subzones in the scale-down strategies study.**

Current release subzones have a total population of 1,304,700.

| **Zone** | **Subzone** | **Population size** |
| --- | --- | --- |
| Tampines | Tampines North | 25680 |
| Tampines | Tampines East | 126460 |
| Tampines | Tampines West | 81370 |
| Woodlands | Midview | 34580 |
| Woodlands | Woodlands East | 100220 |
| Yishun | Yishun West | 52160 |
| Yishun | Yishun Central | 2960 |
| Yishun | Yishun South | 41920 |
| Yishun | Yishun East | 70190 |
| Yishun | Northland | 27280 |
| Sengkang | Sengkang Town Centre | 61210 |
| Sengkang | Rivervale | 59180 |
| Marine Parade - Mountbatten | Mountbatten | 10260 |
| Marine Parade - Mountbatten | Katong | 9450 |
| Marine Parade - Mountbatten | Frankel | 35010 |
| Punggol | Punggol Field | 49010 |
| Hougang | Hougang West | 44840 |
| Holland | Ulu Pandan | 11470 |
| Geylang - MacPherson | MacPherson | 27120 |
| Commonwealth | Holland Drive | 12210 |
| Commonwealth | Mei Chin | 16590 |
| Clementi - West Coast | West Coast | 10100 |
| Clementi - West Coast | Clementi Central | 14780 |
| Clementi - West Coast | Clementi North | 29400 |
| Choa Chu Kang | Yew Tee | 40070 |
| Choa Chu Kang | Peng Siang | 34250 |
| Choa Chu Kang | Keat Hong | 38990 |
| Bukit Merah - Telok Blangah | Alexandra Hill | 13220 |
| Bukit Merah - Telok Blangah | Redhill | 11090 |
| Bukit Merah - Telok Blangah | Henderson Hill | 13000 |
| Bukit Merah - Telok Blangah | Tiong Bahru Station | 14870 |
| Bukit Merah - Telok Blangah | Tiong Bahru | 12240 |
| Bukit Merah - Telok Blangah | Kampong Tiong Bahru | 8840 |
| Bukit Merah - Telok Blangah | Telok Blangah Rise | 11780 |
| Bukit Merah - Telok Blangah | Telok Blangah Way | 8590 |
| Bukit Batok | Hong Kah North | 25660 |
| Bedok | Kaki Bukit | 35880 |
| Bedok | Bedok North | 82770 |

**Table S5. List of new subzones in the redistribution strategies study.**

New release subzones have a total population of 615,130 people.

| **Zone** | **Subzone** | **Population size** |
| --- | --- | --- |
| Jurong West | Yunnan | 66890 |
| Jurong West | Jurong West Central | 62960 |
| Jurong West | Hong Kah | 53380 |
| Jurong West | Boon Lay Place | 28590 |
| Jurong West | Taman Jurong | 39600 |
| Jurong West | Wenya | 8280 |
| Toa Payoh | Toa Payoh Central | 27570 |
| Novena | Balestier | 33040 |
| Kallang | Bendemeer | 37420 |
| Ang Mo Kio | Cheng San | 27280 |
| Ang Mo Kio | Chong Boon | 25680 |
| Ang Mo Kio | Yio Chu Kang West | 23820 |
| Ang Mo Kio | Kebun Bahru | 22250 |
| Ang Mo Kio | Townsville | 20210 |
| Ang Mo Kio | Shangri-La | 17630 |
| Pasir Ris | Pasir Ris West | 35580 |
| Pasir Ris | Pasir Ris Drive | 52650 |
| Pasir Ris | Pasir Ris Central | 32300 |
