# Supplementary material for: Wolbachia incompatible insect technique program optimization over large spatial scales using a process-based model of mosquito metapopulation dynamics
Source: BMC Biol. 2024 Nov 21;22:269. doi: 10.1186/s12915-024-02070-1 (PMC11580355; doi:10.1186/s12915-024-02070-1)

#### **Additional File 5**

**Sensitivity analysis results**

We performed sensitivity analyses of the mean *IE_final_* values for each strategy by varying the key model parameters by 30% above and below the main experiment value. Four parameter values (male mortality, additional *Wolbachia-*infected male mortality, overflooding ratio, and migration rate) were given a wider range of values.


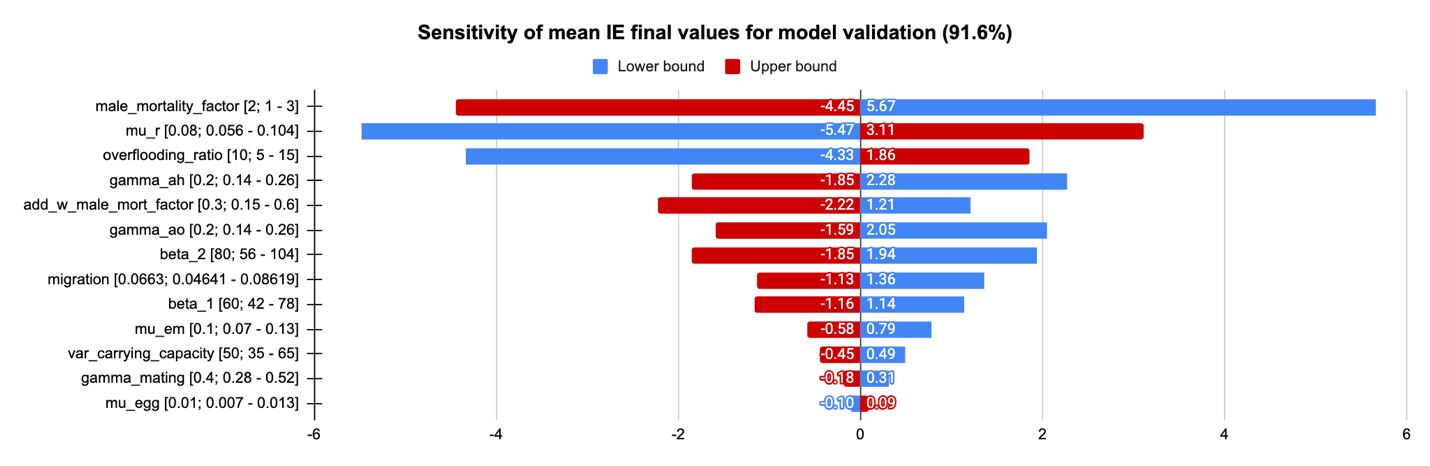

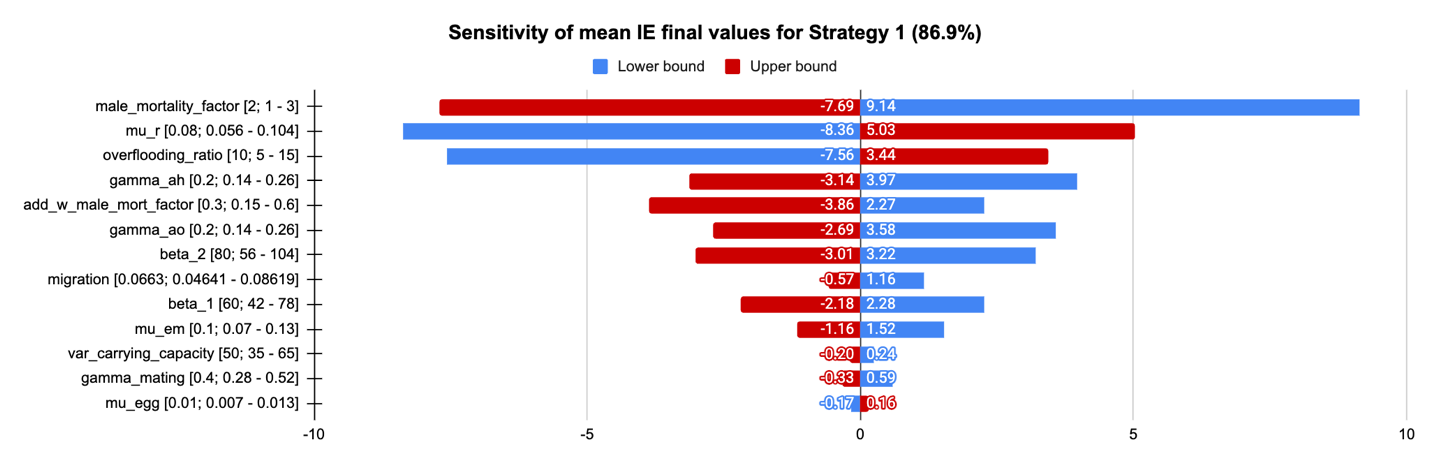

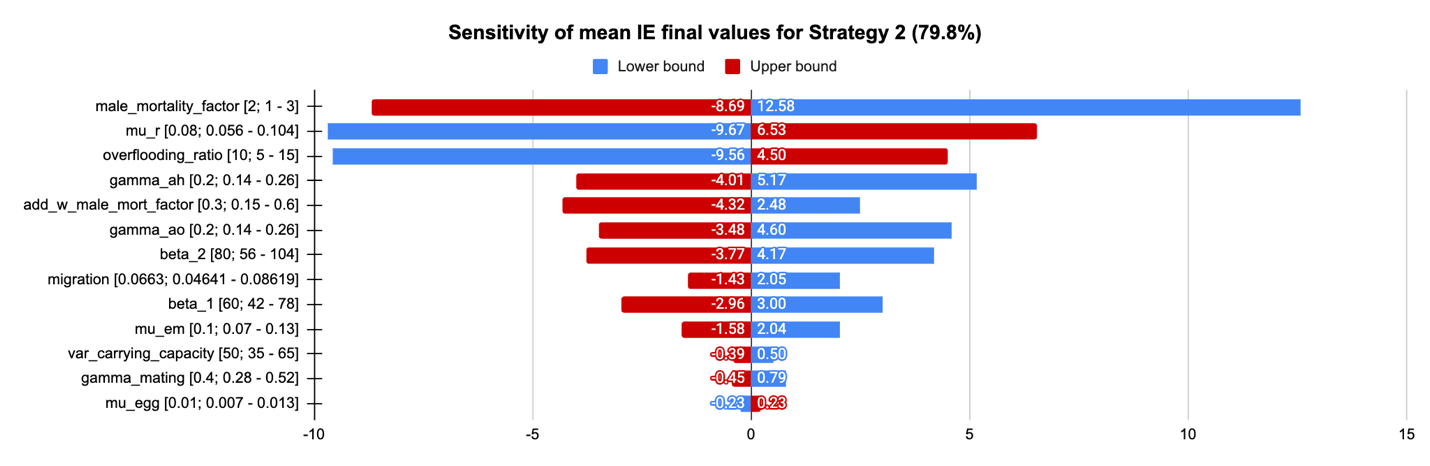

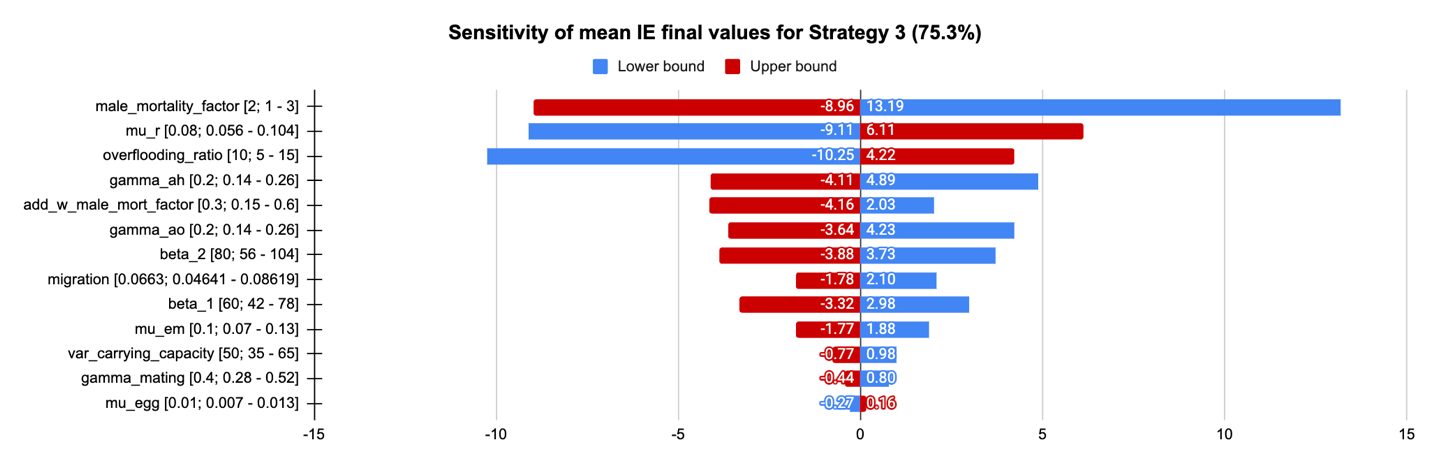

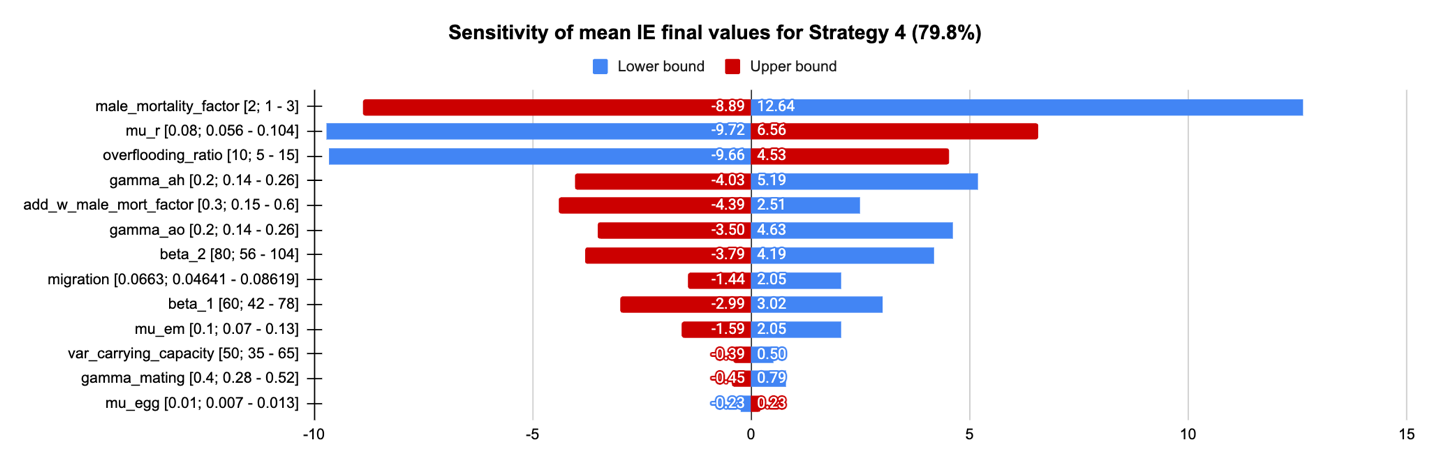

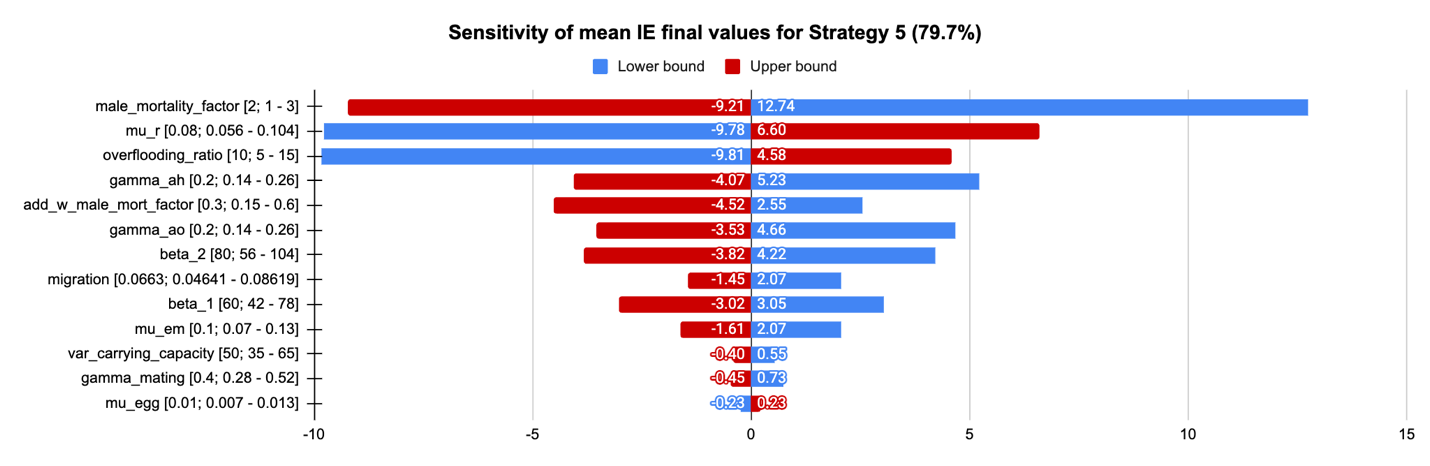

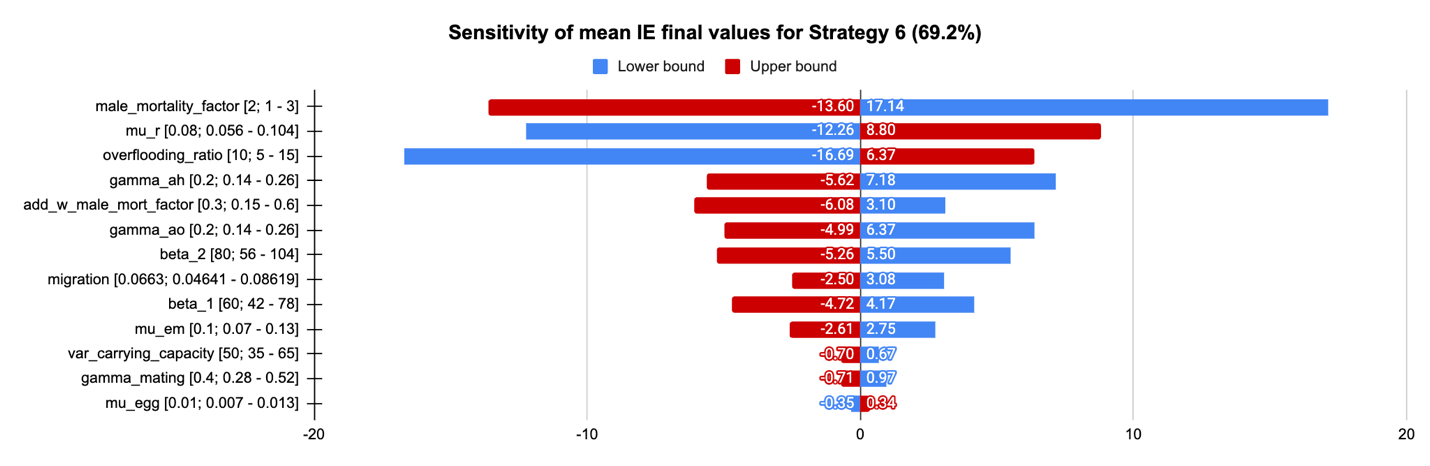

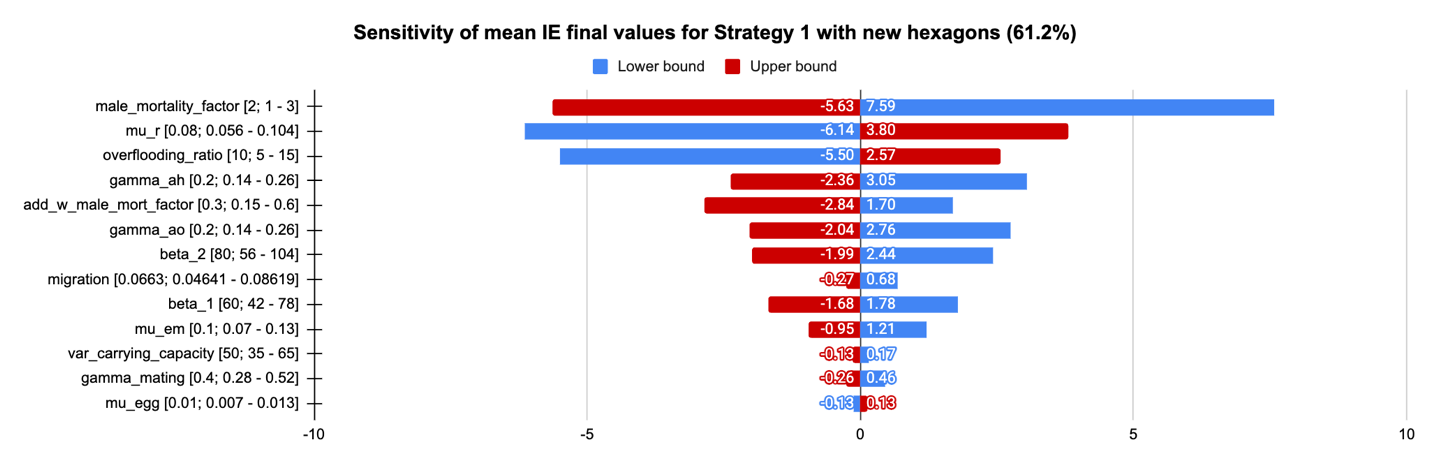

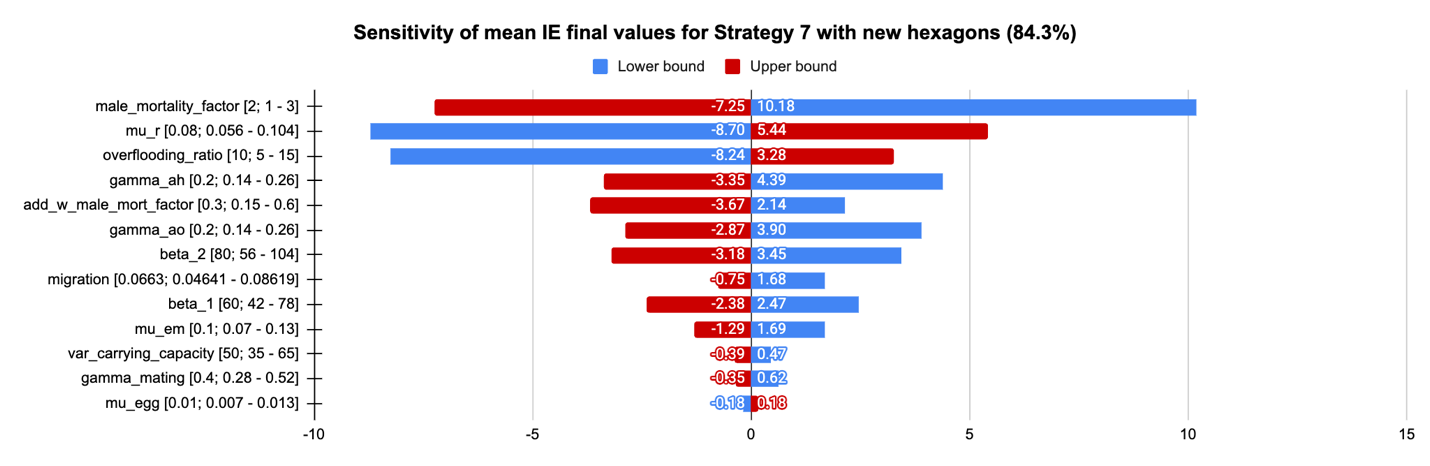

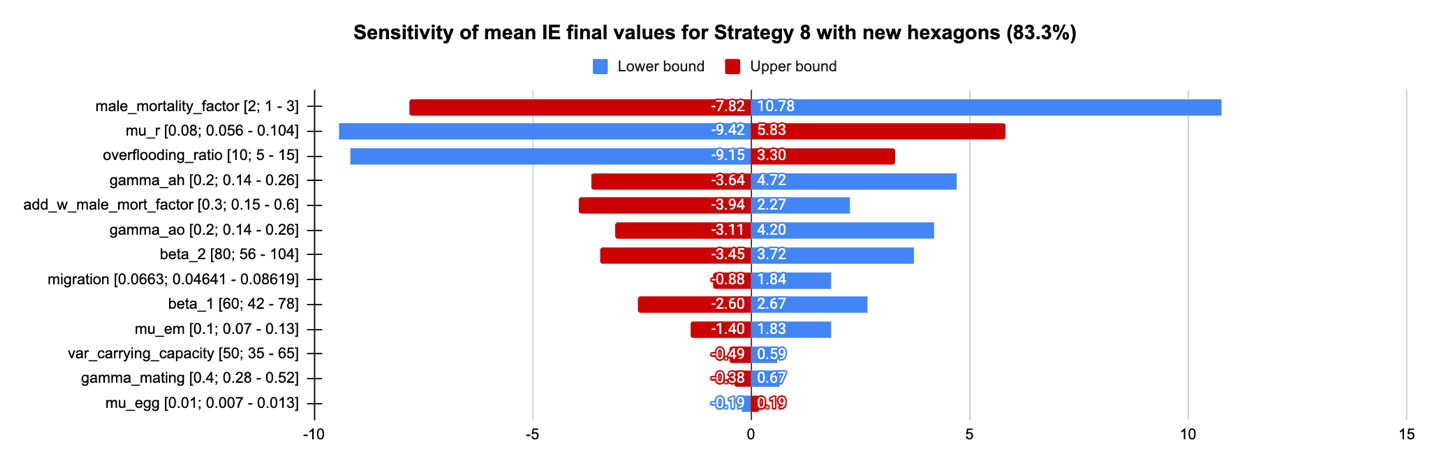

Supplement: Supplementary file 5 — Additional file 5: Sensitivity analysis results. [file 12915_2024_2070_MOESM5_ESM.docx]
